# Nurses’ reported training needs for advanced cell therapies: a survey on behalf of the Nurses Group of the EBMT

**DOI:** 10.46989/001c.124593

**Published:** 2024-11-15

**Authors:** Michelle Kenyon, Sarah Jayne Liptrott, Annika Kisch, Jarl Mooyaart, Brian Piepenbroek, Daphna Hutt, Isabel Salcedo, Annalisa Ruggeri, Cristian Chabannon, Rose Ellard, John Murray

**Affiliations:** 1 Department of Haematological Medicine, 4th Floor Hambledon Wing West, King’s College Hospital NHS Foundation Trust, Denmark Hill, London, United Kingdom; 2 Nursing Development and Research Unit, Oncology Institute of Southern Switzerland, Ente Ospedaliero Cantonale (EOC), via Gallino 12, 6500, Bellinzona, Switzerland; 3 Department of Nursing, Regional Hospital of Bellinzona e Valli, Ente Ospedaliero Cantonale (EOC), via Gallino 12, 6500, Bellinzona, Switzerland; 4 Department of Hematology, Oncology and Radiation Physics, Skane University Hospital, Lund, Sweden; 5 Institute of Health Sciences, Lund University, Lund, Sweden; 6 EBMT Leiden Statistical Unit, Rijnsburgerweg 10, 2333 AA, Leiden, The Netherlands; 7 EBMT Leiden Study Unit, Rijnsburgerweg 10, 2333 AA, Leiden, The Netherlands; 8 Department of Paediatric Hematology-Oncology and BMT, Edmond and lily Safra Children Hospital, Sheba Medical center, Ramat-Gan 5265601 Israel; 9 Hospital Universitario Puerta de Hierro Majadahonda, Spain; Instituto de Investigación Sanitaria Puerta de Hierro-Segovia Arana. Calle Joaquín Rodrigo 2, 28222 Majadahonda, Spain; 10 Dept of Hematology, IRCCS San Raffaele Scientific Institute, 20135 Milano, Italy; 11 Centre de Thérapie Cellulaire. Institut Paoli-Calmettes Comprehensive Cancer Centre & module Biothérapies du Centre d’Investigations Cliniques de Marseille, Inserm CBT-1409, Aix-Marseille Université, AP-HM, Institut Paoli-Calmettes. Marseille, France; 12 The Royal Marsden NHS Foundation Trust, London, UK; 13 Haematology and Transplant Unit The Christie NHS FT, Manchester, United Kingdom

**Keywords:** ATMP, CAR-T, cellular therapy, gene therapy, nursing care, education

## Abstract

**Background:**

Advanced Therapy Medicinal Products (ATMPs) for human use have advanced globally with the rapid adoption of Chimeric Antigen Receptor T-cell (CAR-T) therapies in haemato-oncology. CAR-T cell therapy and ATMPs have unique, significant acute and chronic toxicities, and appropriate patient care is crucial. Significant challenges, including the need for nurse education and training, accompany optimal patient success and benefits.

**Objectives:**

This study aimed to describe nurses’ training needs in relation to ATMP management and patient care.

**Methods:**

A cross-sectional online survey was performed by the European Society for Blood and Marrow Transplantation, based on a previously tested questionnaire developed in the UK.

**Findings:**

109 complete responses from 86 different centers from 24 countries were returned (1207 distributed). Over 1/3 reported experience delivering licensed ATMPs (CAR-T). High-priority training areas included a general introduction to ATMPs, toxicity management, product-specific information, and regulatory frameworks for ATMPs. A clear need for ATMP-specific training exists and is regarded as important. Training prior to implementation is key and should be supported by ongoing competency maintenance. Counseling, patient support, and long-term follow-up are identified for future training and opportunities for nurse experience sharing in this rapidly evolving field.

## Introduction

Advanced Therapy Medicinal Products (ATMPs), also referred to as advanced cell therapies, are medicines for human use based on genes, cells, or tissue engineering. Over recent years, global advances in this area have been significant.^1^

In Haemato-oncology, the swift adoption of Chimeric Antigen Receptor T-cell (CAR-T) therapies herald the first commercial success of industry-manufactured ATMPs on a worldwide scale. Autologous CAR-T products targeting CD19 were approved in Europe for relapsed/refractory B-cell acute lymphoblastic leukemia, high-grade B-cell lymphoma, and mantle cell lymphoma.^2^ The numbers of approved products and indications have significantly increased since the original approvals of tisacel and axicel in mid-2018, and so have the numbers of treated patients. Nevertheless, the actual fraction of patients who may benefit from receiving and who do receive CAR-T remains largely unknown. To achieve full success for the benefit of patients and families, formidable challenges must be tackled. In particular, qualification of a growing number of EU hospitals has been accompanied by demand for information, education and training, in order to equip personnel with the skills required to deliver ATMPs and provide appropriate patient care at the bedside.^3,4^ Nurses play a crucial role in assessing and monitoring patients throughout this pathway, and nurse education and knowledge are fundamental to this.

In haemato-oncology, ATMPs of hematopoietic origin or nature have often been delivered alongside adult and pediatric haematopoietic stem cell transplant (HSCT) programs in the early years after the first approvals. The procurement, manufacture, administration, and follow-up processes all differ from those associated with routine stem cell transplantation, and even further from administration of any other category of drug or drug combination.^5^

Furthermore, CAR-T cell therapy and ATMPs more generally bear their own unique set of significant acute and chronic toxicities ranging from mild to lethal. The most commonly observed acute toxicity, cytokine release syndrome (CRS), usually manifests initially as fever, hypoxia and hypotension, and may be accompanied by rigors, malaise, headaches, myalgias, arthralgias and anorexia, which can progress rapidly to life threatening capillary leak syndrome and tachycardia.^6^ Neurological toxicity is the second major side effect and, although typically self-limited, it can be potentially life threatening or fatal.^7^ Beyond the immediate post-infusion phase, B-cell aplasia, hypogammaglobulinemia, chronic cytopenias and resulting infections pose the main risks, with secondary malignancies rarely reported.^8–11^ These toxicities require acquisition of expert nursing knowledge and care since they are rarely experienced in other contexts.^2,12^

Based on the need for nurses to be adequately informed and competent in ATMP administration, the purpose of this study was to understand the current level of training and further determine education needs of adult and pediatric nurses working in centers currently delivering or aiming to deliver ATMPs.

## Materials and Methods

### Study design

A cross-sectional survey was distributed online to EBMT NG national chairs, nurse contacts of the IEC (immune effector cell) forum, EBMT center principal investigators and coordinators, and any other centers registered through EBMT as delivering licensed and investigational products. Identified participants were emailed a link to the survey which was uploaded to the ‘SurveyMonkey’ platform. The email highlighted that survey participation was voluntary and that consent was assumed upon completion of the survey. All survey items required response to allow submission. The survey was opened on 29 September 2020 for 4 months, with prompts sent to non-responders at 4, 8 and 12 weeks after which the survey was closed.

### Data collection

The survey was based on one previously developed, tested, and implemented in the UK to determine training needs of adult and pediatric nurses working in haemato-oncology centers delivering either licensed and/or unlicensed ATMPs (IEC/CAR-T therapy).^13^ It was modified to reflect changes in the field since the original survey was devised and wording changed for an international survey. Permissions were sought and agreed from the original authors.

The survey included 61 questions, collecting demographic data including center characteristics, current area of practice, patient age group treated, role and years of experience in hematology or transplantation. Questions also investigated experience with ATMPs, training level, local procedures, organizational structure, and self-assessed priority ranking for ATMP education. All data collection was performed by the Leiden Study Unit according to EBMT guidelines.

### Data Analysis

Survey responses were analyzed using descriptive statistics. Percentages are reported excluding missing responses. A response is considered missing when nothing has been filled in by the center, hence answers such as “Don’t know” and “Not applicable” are considered valid responses. 

## Results

Of the 1207 surveys sent out, 147 were received. After excluding 38 responses with incomplete answers on key variables (e.g. demographics and training availability), a total of 109 responses from 86 different centers from 24 countries were included in the analysis. Characteristics of respondents and treating centers are reported in Table 1. The majority of respondents worked in JACIE accredited (n=78, 72%), and academic centers (n=95, 87%), with adult patients (n=66, 61%). Specialist nurses (n=59, 54%) or registered nurses (n=26, 24%) made up the greatest number of respondents with over two-thirds (n=74, 68%) having more than 10 years’ experience in haematology and HSCT. The majority of respondents said that ATMPs were either being delivered at their center (n=75, 69%), or would be in the near future (n=13, 12%). Many respondents had no experience with licensed or unlicensed ATMPs (n=23, 21%; n=32, 30%; respectively). Many respondents reported how their center was involved in the follow-up of patients who had received ATMPs at another center (n=35, 32%), or would be following these patients in the near future (n=11, 10%).

**Table 1.** Demographics

	*N Missing*	*N = 109*
JACIE accredited	0	
No		11 (10%)
Yes		78 (72%)
In process/Pending		15 (14%)
Don’t know		5 (4.6%)
**Academic centre**	0	95 (87%)
**Type of patients**	0	
Children (0-18 years)		30 (28%)
Adults (>18 years)		66 (61%)
Both		13 (12%)
**Practice area: Day Hospital**	0	28 (26%)
**Practice area: Bone Marrow Transplant In-Patient Unit**	0	90 (83%)
**Practice area: Bone Marrow Transplant Out-Patient Unit**	0	58 (53%)
**Practice area: Other**	0	28 (26%)
**Role**	0	
Registered Nurse		26 (24%)
Research Nurse		9 (8.3%)
Ward Manager		12 (11%)
Specialist Nurse		59 (54%)
Quality Manager		3 (2.8%)
**Years experience in Haematology/BMT**	0	
0 - 2 years		4 (3.7%)
2 - 5 years		7 (6.4%)
5 - 10 years		24 (22%)
10 - 15 years		26 (24%)
> 15 years		48 (44%)
**Experience delivering unlicensed advanced therapies (ATMPs)**	4	
None		32 (30%)
Some		43 (41%)
Experienced		23 (22%)
Don’t know		7 (6.7%)
**Experience delivering licensed advanced therapies (ATMPs)**	2	
None		23 (21%)
Some		37 (35%)
Experienced		41 (38%)
Don’t know		6 (5.6%)
**Delivery of ATMP at your centre**	0	
No		21 (19%)
Yes		75 (69%)
Not yet, but in the near future		13 (12%)
**Follow-up of patients at other centre**	0	
No		63 (58%)
Yes		35 (32%)
Not yet, but in the near future		11 (10%)

ATMPs – Advanced Therapy Medicinal Products; BMT – Bone Marrow Transplant; JACIE - Joint Accreditation Committee of the International Society for Cell and Gene Therapy and the European Group for Blood and Marrow Transplantation

Respondents were asked to reflect on their current areas of knowledge and identify whether training on specific topics in relation to ATMPs was available, received or required, being developed or not available. Over half of respondents had received or had training available regarding a general overview of ATMPs, ATMP manufacturing, handling and principles of cryopreservation and transportation of cell products (Table 2). Half had received or had access to training regarding individual cell types (e.g., dendritic cells, tumor infiltrating lymphocytes, mesenchymal stem cells, etc.) used in ATMPs (n=54, 50%).

**Table 2.** Training availability (N=109)

	*Available*	*Not available*	*Not applicable*	*Missing*
**General:**				
General introduction to ATMPs	84 (77%)	25 (23%)	0 (0%)	0 (0%)
A general overview of ATMP manufacturing	63 (58%)	46 (42%)	0 (0%)	0 (0%)
ATMP handling, transfer and follow-up traceability	65 (60%)	44 (40%)	0 (0%)	0 (0%)
Educational resources on individual cell types	54 (50%)	55 (50%)	0 (0%)	0 (0%)
Principles of cryopreservation and transportation	75 (69%)	34 (31%)	0 (0%)	0 (0%)
**Procedure:**				
Assessing the appearance of a cell-based product	69 (63%)	38 (35%)	0 (0%)	2 (1.8%)
Disposal of ATMP residue and packaging, gene products	60 (55%)	47 (43%)	1 (0.9%)	1 (0.9%)
Disposal of ATMP residue and packaging, non-gene products	62 (57%)	45 (41%)	1 (0.9%)	1 (0.9%)
Local procedure for ordering ATMP X from manufacturer Y	62 (57%)	44 (40%)	3 (2.8%)	0 (0%)
Local procedure for taking receipt of an ATMP	68 (62%)	38 (35%)	3 (2.8%)	0 (0%)
Managing ATMP spills	62 (57%)	47 (43%)	0 (0%)	0 (0%)
Thawing a cryopreserved cell-based product	73 (67%)	35 (32%)	1 (0.9%)	0 (0%)
**CAR-T management:**				
CAR-T toxicity management: CRS	79 (72%)	29 (27%)	1 (0.9%)	0 (0%)
CAR-T toxicity management: ICANS	77 (71%)	31 (28%)	1 (0.9%)	0 (0%)
CAR-T toxicity management: others	75 (69%)	32 (29%)	1 (0.9%)	1 (0.9%)
Counselling / support for patient and carers	60 (55%)	49 (45%)	0 (0%)	0 (0%)
Local regulatory framework for ATMPs	59 (54%)	49 (45%)	1 (0.9%)	0 (0%)
Long Term Follow-Up of patients	59 (54%)	48 (44%)	2 (1.8%)	0 (0%)

ATMPs – Advanced Therapy Medicinal Products; CAR-T - Chimeric Antigen Receptor T-cell therapies; CRS - Cytokine Release Syndrome; ICANS - Immune effector Cell-Associated Neurotoxicity Syndrome

As for local procedures related to ATMPs, the availability of training or training having been received was reported by over half of respondents in relation to cell product appearance (n=69, 63%), gene and non-gene product residue/packing disposal (n=60, 55%; n=62, 57%), ordering ATMPs (n=62, 57%), taking receipt of ATMPs (n=68, 62%), and managing ATMP spills (n=73; 67%). Slightly more common was training on thawing of a cell-based product (n=73, 63%) (Table 2).

Training for CAR-T toxicity management (including CRS and ICANS) was much more commonly available (n=79, 72%; n=77, 71%), while training surrounding the local regulatory framework for ATMPs and patient management in terms of counselling/support and long-term follow-up was less commonly available (n=59, 54%; n=60, 55%; n=59, 54%) (Table 2).

When asked about training priorities for resource development, all suggested topics were given a medium/high priority, in particular a general introduction to ATMPs (n=104, 95%), CAR-T toxicity management (n=103, 94%), drug products used in ATMPs (n=103, 94%) and the local regulatory framework for ATMPs (n=102, 94%). The lowest priority was given to the local procedure for ordering ATMP from manufacturers (n=22, 20%) (Table 3).

**Table 3.** Training priorities (N=109)

	*Medium/high priority*	*Low priority*	*Missing*
**Training priorities:**			
ATMP chain of identity and chain of custody	89 (82%)	18 (17%)	2 (1.8%)
CAR-T toxicity management	103 (94%)	3 (2.8%)	3 (2.8%)
Disposal of ATMP (e.g. CAR-T) residue and packaging	100 (92%)	7 (6.4%)	2 (1.8%)
General introduction to ATMPs	104 (95%)	3 (2.8%)	2 (1.8%)
Individual drug products used in ATMPs	103 (94%)	3 (2.8%)	3 (2.8%)
Local procedure for ordering ATMP X from manufacturer Y	85 (78%)	22 (20%)	2 (1.8%)
Local procedure for taking receipt of an ATMP	90 (83%)	17 (16%)	2 (1.8%)
Local regulatory framework for ATMPs	102 (94%)	5 (4.6%)	2 (1.8%)
Managing ATMP (e.g. CAR-T) spills	100 (92%)	7 (6.4%)	2 (1.8%)
Overview of ATMP manufacturing, introducing concepts/terminology	94 (86%)	11 (10%)	4 (3.7%)
Principles of cryopreservation and cryotransportation	90 (83%)	17 (16%)	2 (1.8%)
Thawing a cryopreserved cell-based product	94 (86%)	13 (12%)	2 (1.8%)
Visual inspection of a cell-based product	98 (90%)	9 (8.3%)	2 (1.8%)

ATMPs – Advanced Therapy Medicinal Products; CAR-T - Chimeric Antigen Receptor T-cell therapies

For respondents who had already worked with ATMPs before (n=90), areas where they felt that staff would have benefited from further education and training included a basic overview (n=19, 21%), toxicity and side effects (n=17, 19%) and continuous advanced overview (n=15, 17%) (Table 4). Over half of these respondents also reported having staff in the hospital working in roles that specifically provided staff education and training (n=52, 58%). Among the respondents that reported on which staff provided education and training (n=37) predominantly nurses in practice development/ practice education roles (45.9%) or nurses in advanced practice roles (18.9%) were indicated.

**Table 4.** Education that would have been beneficial for staff

	*N = 90*
Basic overview	19 (21%)
Toxicity and side effects	17 (19%)
Infusion problems	3 (3.3%)
Discharge care	1 (1.1%)
Long-term follow-up	6 (6.7%)
COVID-19	1 (1.1%)
Continuous/advanced overview	15 (17%)
Supportive care patient/family	9 (10%)

The majority of respondents who had worked with ATMPs had received product specific training from pharmaceutical companies regarding ATMPs (n=58, 64.0%) and, in a small number of cases (n=7), discrepancies were identified between pharmaceutical company and local procedures. Examples included ATMP administration, patient monitoring, and side effect management, which was reported to make the development of Standard Operating Procedures a challenge.

Respondents were asked whether they were members of working groups related to advanced therapies and, while the majority were not (n=54, 64.0%), there was evidence of networking with membership in local /regional groups (n=2, 2.4%), national groups (n=19, 23.0%) and international groups (n=5, 6.0%).

## Discussion

Advanced Therapy Medicinal Products represent a significant breakthrough in the field of hematology and HSCT. They offer new opportunities for the treatment of disease and injury^14^ and nurses need to be skilled in their delivery and have access to suitable and appropriate teaching and training materials.

Hematology and transplant nurses are well versed in the requirement to satisfy training and competency knowledge and have complied with the Foundation for the Accreditation of Cellular Therapy (FACT) and Joint Accreditation Committee International Society for Cell and Gene Therapy (ISCT)-Europe & EBMT (JACIE). This includes the administration of HSC, IEC, genetically modified and other cellular therapies. Meeting FACT-JACIE standards^15^ and being accredited lead to the delivery of high-quality patient care, and can only be achieved by having a well-trained and educated work force. However, in order to respond to nurses needs, it is important to establish what is available and which learning needs must be addressed.

Although the use of ATMPs is relatively recent, the majority of respondents in this survey reported that their centers were involved in delivering ATMPs or would be doing so in the near future, a finding that reflects the diffusion of ATMPs in the clinical setting and growing indications.^16^ A minority of respondents in this survey indicated having no experience with licensed or unlicensed ATMPs, a finding that may be challenged today as the experience of nurses may have increased significantly. Interestingly, one third of respondents reported providing follow-up care for patients receiving ATMPs in another center. This reflects models of care, where advanced medical services and complex treatments are delivered by centralized services, while more basic services such as follow-up care are delivered via secondary sites.^17^ All healthcare professionals caring for patients receiving ATMPs require education and training, and this should not be limited to those only involved in treatment delivery.

ATMP is a growing market and, for nurses, this change in therapy is accompanied by the need for additional training that ranges from basic to advanced, depending upon the center and country experience. Many of the key training topics suggested in this survey were reported to be either available or have been received for over half of respondents, in particular that of cell thawing, which is a standard procedure in autologous SCT and, therefore, may be well established as part of existing training.^15^

The availability of training regarding toxicities and their management was particularly evident within this group of respondents, a finding which is not surprising, as toxicities relating to ATMPs can be novel, and range from mild to life threatening.^12^ Nurses have a key role in identifying adverse events and prompting management by the healthcare team. JACIE standards require nurses to have specific training in the complications associated with cellular therapy administration, such as CRS.^15^ There were fewer reports of training opportunities surrounding patient counselling/support and long-term follow-up, perhaps as this is a relatively new field of care.

When asked what the priorities were for resource development, areas that were ranked as having a higher training priority were visual inspection and the management of ATMP spills, basic and procedural knowledge, and patient management. These suggestions reflect the need for support in areas where nurses have an active role and responsibility. Areas that were ranked as a low training priority included cryopreservation and thawing, chain of identity/custody and local procedures for ordering or taking receipt of an ATMP – again areas which are perhaps less commonly perceived as direct nursing care activities. These are all aspects that are vital in the safe delivery of ATMP and require a good understanding of process and procedure.

Providing education and facilitating competency development is a key component of some nursing roles, particularly practice educators and practice development nurses. ATMP handling and patient care is currently a niche area which may account for staff education and training being provided by specialist nurses, physicians, and CAR-T coordinators. Training in almost two-thirds of cases was also provided by pharmaceutical companies, which reflects requirements as part of risk mitigation activities to ensure appropriate qualification and preparation of the center.^18^ While standard operating procedures can facilitate a standardized approach to procedures and patient care, conflict can arise where discrepancies are experienced between pharmaceutical company requirements and those of the local center – as reported by some respondents in this study. Open discussions that include all stakeholders such as the GoCART Coalition are required to find solutions to such issues while ensuring patient safety. GoCART is an EBMT multi-stakeholder coalition of patient representatives, health care professionals, pharmaceutical companies, regulators, health technology assessment (HTA) bodies and reimbursement agencies, and medical organisations, collaborating to maximise the potential of cellular therapies manufactured from cells and tissues of hematopoietic origin.

This EBMT NG study of nurses’ perceptions of their training needs for advanced therapy medicinal products like CAR-T therapy suggests that there is a benefit from opportunities to share experiences and receive more nursing-centered education, especially with regards to therapy side effects and patient management e.g., patient/family education, expectation management, and supportive care. Education strategies should have a broad scope in order to incorporate the needs of the various complex roles that nurses are engaged in across the ATMP patient pathway. To deliver safe and effective care, nurse education must be of the highest quality and readily available.

It is suggested that different approaches should be employed to educate staff, based on the topic, required outcome and principles of adult learning,^19^ identifying issues more appropriate for didactic or practical methods. Several resources exist that can support the development of education for staff in this setting. Hayden et al.^2^ provides best practice recommendations from key hematology/transplant groups regarding patient management, including work-up, apheresis, pre-treatment therapies, reinfusion and complications – written by a variety of healthcare professionals. Nursing specific guidelines have been written including a general introduction to CAR-T therapy, aspects of apheresis and specifics of nursing care during the infusion, supportive therapies, managing infusion reactions, toxicities and follow-up of patients.^12^ These structured documents can be used to provide local guidance in practice. Other nursing resources include the CAR-T therapy booklet and e-learning programs, describing an overview of the immune system and adoptive cell transfer, the autologous CAR-T process, treatment administration, follow-up and psychological implications as well as aspects specific to a pediatric population.^20^ Another resource is the IEC forum which provides space for nurses and allied healthcare professionals to post questions and initiate dialogues about practice-promoting education and information exchange.^21^ The Advanced Therapy Treatment Centres Network^22^ provides a range of resources including case studies, manufacturing and preparation toolkits, readiness toolkits and a range of webinars and videos sharing experiences and providing some ‘real life’ experiences to help with critical thinking.

Developing education to address nurses’ needs is also context dependent. Centers with well-established HSCT programs may find it easier to incorporate ATMP education programs within existing training. Here, the infrastructure that supports HSCT from a regulatory and cell processing perspective will already be in place, as will the care delivery program.^19^ The challenge is that, as the ATMPs scope widens and extended to the treatment of patients with solid organ diseases,i.e., non-hematological conditions, if care is to be delivered within a hematology/transplant department setting, the nursing team will need additional support and training to broaden their knowledge base to adequately care for this new patient population.^19^ On the other hand, with increasing developments in CAR-T therapy in solid tumors,^23^ auto-immune diseases, inflammatory conditions and degenerative diseases, HSCT programs may not have the capacity to treat all patients and, as such, innovative solutions to ATMP delivery and training for staff in non-specialized centers is a likely reality. Other centers such as the Seattle Cancer Care Alliance maintain a separate CAR-T program to centralize clinical care and staff education specific to the unique complications of this treatment.^19^ Continuing cyclical education programs that offer updates on latest practice and evidence-based care are firmly embedded in transplant accredited centers, with those such as City of Hope developing a robust quality management system with a checklist that allows regular quarterly reviews of training compliance.^24^ Periodic training and updates will surely be a support for managing patients in such a rapidly developing context.

It is acknowledged that there are some limitations with the survey. Some time has passed since the survey was performed, which may suggest that findings could be different today in the light of training having been developed, more ATMPs being used and greater familiarity with the products. A further study would be helpful to investigate changes in this setting also in light of the number of respondents who suggested they would be involved in ATMP delivery in the near future. However, factors such as nursing staff turnover intimate the continuing need for basic training and refresher training for those delivering ATMPs in smaller numbers. Increasing numbers of products and indications also suggest that service capacity constraints may drive the need to deliver products in centers not usually administering ATMPs, and, as such, training needs to be accessible to all. The total number of responses to the survey was low, suggesting the results cannot be generalized. Thus, making assumptions regarding the education needs of centers that did not respond is difficult. One limitation of the survey was that it was only available in English. Despite prior use of the survey and rewording of some questions for an international context, it is possible that some participants misinterpreted or misunderstood some of the questions. Future surveys should be available in a range of languages to allow for greater reach and facilitate the completion of the questionnaire.

In conclusion, our findings reveal a clear need for nursing-centered ATMP education and training. Before implementation, education should be provided for each advanced therapy and should cover general knowledge as well as specific training on patient management, visual inspection of the product, management of spills, and therapy side effects. Counseling, support, and long-term follow-up remain areas for training development. Importantly, the self-expressed need to share nursing experiences identified via our survey offers future collaborative projects and knowledge consolidation opportunities.

### Authors’ Contribution

Conceptualization: Michelle Kenyon (Equal), Rose Ellard (Equal), John Murray (Equal). Formal Analysis: Michelle Kenyon (Equal), Sarah Jayne Liptrott (Equal), Annika Kisch (Equal), Jarl Mooyaart (Equal), Brian Piepenbroek (Equal), John Murray (Equal). Investigation: Michelle Kenyon (Equal), Sarah Jayne Liptrott (Equal), Annika Kisch (Equal), Jarl Mooyaart (Equal), Brian Piepenbroek (Equal), John Murray (Equal). Visualization: Michelle Kenyon (Equal), Sarah Jayne Liptrott (Equal), Annika Kisch (Equal), Brian Piepenbroek (Equal), John Murray (Equal). Writing – original draft: Michelle Kenyon (Equal), Sarah Jayne Liptrott (Equal), Annika Kisch (Equal), Jarl Mooyaart (Equal), Brian Piepenbroek (Equal), John Murray (Equal). Writing – review & editing: Michelle Kenyon (Equal), Sarah Jayne Liptrott (Equal), Annika Kisch (Equal), Jarl Mooyaart (Equal), Brian Piepenbroek (Equal), Daphna Hutt (Equal), Isabel Salcedo (Equal), Annalisa Ruggeri (Equal), Cristian Chabannon (Equal), Rose Ellard (Equal), John Murray (Equal). Methodology: Sarah Jayne Liptrott (Equal), Annika Kisch (Equal), Isabel Salcedo (Equal). Project administration: Sarah Jayne Liptrott (Equal), Annika Kisch (Equal), Brian Piepenbroek (Equal), Isabel Salcedo (Equal). Supervision: Sarah Jayne Liptrott (Equal), Annika Kisch (Equal). Data curation: Jarl Mooyaart (Equal), Brian Piepenbroek (Equal). Resources: Jarl Mooyaart (Equal), Brian Piepenbroek (Equal). Software: Jarl Mooyaart (Equal), Brian Piepenbroek (Equal). Validation: Jarl Mooyaart (Lead).

### Conflict of Interest – COPE

Michelle Kenyon: Mallinkrodt, Jazz, Sanofi

Sarah Jayne Liptrott: No competing interests were disclosed.

Annika Kisch: No competing interests were disclosed.

Jarl Mooyaart: No competing interests were disclosed.

Brian Piepenbroek: No competing interests were disclosed.

Daphna Hutt: No competing interests were disclosed.

Isabel Salcedo: No competing interests were disclosed.

Annalisa Ruggeri: No competing interests were disclosed.

Christian Chabannon: Bellicum Pharmaceuticals, BMS, Jazz, Janssen Pharmaceuticals, Kite / Pharma, Novartis, Sanofi SA

Rose Ellard: Kite Gilead, Novartis, Janssen, BMS Celgene.

John Murray: Therakos, Jazz Pharmaceuticals, Janssen and Gilead/Kite

### Ethical Conduct Approval – Helsinki – IACUC

(for more information, read https://chi.scholasticahq.com/for-authors)

Ethical approval for the survey was not sought. The email highlighted that survey participation was voluntary and that consent was assumed upon completion of the survey.

### Informed Consent Statement

All authors and institutions have confirmed this manuscript for publication.

### Data Availability Statement

All are available upon reasonable request.

## References

[ref-368148] Passweg J. R., Baldomero H., Chabannon C., Basak G. W., de la Cámara R., Corbacioglu S., Dolstra H., Duarte R., Glass B., Greco R., Lankester A. C., Mohty M., Peffault de Latour R., Snowden J. A., Yakoub-Agha I., Kröger N., European Society for Blood and Marrow Transplantation (EBMT) (2021). Hematopoietic cell transplantation and cellular therapy survey of the EBMT: monitoring of activities and trends over 30 years. Bone marrow transplantation.

[ref-368144] Hayden P. J., Roddie C., Bader P., Basak G. W., Bonig H., Bonini C., Chabannon C., Ciceri F., Corbacioglu S., Ellard R., Sanchez-Guijo F., Jäger U., Hildebrandt M., Hudecek M., Kersten M. J., Köhl U., Kuball J., Mielke S., Mohty M., Murray J., Yakoub-Agha I. (2022). Management of adults and children receiving CAR T-cell therapy: 2021 best practice recommendations of the European Society for Blood and Marrow Transplantation (EBMT) and the Joint Accreditation Committee of ISCT and EBMT (JACIE) and the European Haematology Association (EHA). Annals of oncology: official journal of the European Society for Medical Oncology.

[ref-368147] Lowdell M. W., Thomas A. (2017). The expanding role of the clinical haematologist in the new world of advanced therapy medicinal products. British journal of haematology.

[ref-368134] Calmels B., Kröger N., Gribben J. G., Chabannon C., Yakoub-Agha I., Hermann E. (2022). The EBMT/EHA CAR-T Cell Handbook.

[ref-368149] Pillai M., Davies M. M., Thistlethwaite F. C. (2020). Delivery of adoptive cell therapy in the context of the health-care system in the UK: challenges for clinical sites. Therapeutic advances in vaccines and immunotherapy.

[ref-368153] Shimabukuro-Vornhagen A., Gödel P., Subklewe M., Stemmler H. J., Schlößer H. A., Schlaak M., Kochanek M., Böll B., von Bergwelt-Baildon M. S. (2018). Cytokine release syndrome. Journal for immunotherapy of cancer.

[ref-368152] Santomasso B., Bachier C., Westin J., Rezvani K., Shpall E. J. (2019). The Other Side of CAR T-Cell Therapy: Cytokine Release Syndrome, Neurologic Toxicity, and Financial Burden. American Society of Clinical Oncology educational book. American Society of Clinical Oncology. Annual Meeting.

[ref-368136] Cordeiro A., Bezerra E. D., Hirayama A. V., Hill J. A., Wu Q. V., Voutsinas J., Sorror M. L., Turtle C. J., Maloney D. G., Bar M. (2020). Late Events after Treatment with CD19-Targeted Chimeric Antigen Receptor Modified T Cells. Biology of blood and marrow transplantation: journal of the American Society for Blood and Marrow Transplantation.

[ref-368146] Locke F. L., Ghobadi A., Jacobson C. A., Miklos D. B., Lekakis L. J., Oluwole O. O., Lin Y., Braunschweig I., Hill B. T., Timmerman J. M., Deol A., Reagan P. M., Stiff P., Flinn I. W., Farooq U., Goy A., McSweeney P. A., Munoz J., Siddiqi T., Chavez J. C., Neelapu S. S. (2019). Long-term safety and activity of axicabtagene ciloleucel in refractory large B-cell lymphoma (ZUMA-1): a single-arm, multicentre, phase 1-2 trial. The Lancet. Oncology.

[ref-368150] Rejeski K., Subklewe M., Aljurf M., Bachy E., Balduzzi A., Barba P., Bruno B., Benjamin R., Carrabba M. G., Chabannon C., Ciceri F., Corradini P., Delgado J., Di Blasi R., Greco R., Houot R., Iacoboni G., Jäger U., Kersten M. J., Mielke S., Yakoub-Agha I. (2023). Immune effector cell-associated hematotoxicity: EHA/EBMT consensus grading and best practice recommendations. Blood.

[ref-368155] Verdun N., Marks P. (2024). Secondary Cancers after Chimeric Antigen Receptor T-Cell Therapy. The New England journal of medicine.

[ref-368139] Ellard R., Kenyon M., Hutt D., Aerts E., de Ruijter M., Chabannon C., Mohty M., Montoto S., Wallhult E., Murray J. (2022). The EBMT Immune Effector Cell Nursing Guidelines on CAR-T Therapy: A Framework for Patient Care and Managing Common Toxicities. Clinical hematology international.

[ref-368151] Roberts C., Hollingsworth I. (2020). The UK training landscape in advanced therapies for the NHS.

[ref-368141] European Medicines Agency (2023). Advanced therapy medicinal products: Overview.

[ref-368142] Foundation for the Accreditation of Cellular Therapy (FACT), Joint Accreditation Committee – ISCT and EBMT (JACIE) (2021). International Standards for hematopoietic cellular therapy product collection, processing, and Administration.

[ref-368145] Horgan D., Metspalu A., Ouillade M. C., Athanasiou D., Pasi J., Adjali O., Harrison P., Hermans C., Codacci-Pisanelli G., Koeva J., Szucs T., Cursaru V., Belina I., Bernini C., Zhuang S., McMahon S., Toncheva D., Thum T. (2020). Propelling Healthcare with Advanced Therapy Medicinal Products: A Policy Discussion. Biomedicine hub.

[ref-368140] Elrod J. K., Fortenberry J. L., Jr (2017). The hub-and-spoke organization design: an avenue for serving patients well. BMC health services research.

[ref-368133] Berg P., Schönefeld S., Ruppert-Seipp G., Funk M. B. (2022). Regulatory Measures to Improve the Safety of CAR-T-Cell Treatment. Transfusion medicine and hemotherapy: offizielles Organ der Deutschen Gesellschaft fur Transfusionsmedizin und Immunhamatologie.

[ref-368154] Taylor L., Rodriguez E. S., Reese A., Anderson K. (2019). Building a Program: Implications for Infrastructure, Nursing Education, and Training for CAR T-Cell Therapy. Clinical journal of oncology nursing.

[ref-368143] Haematology Nurses and Healthcare Professionals (HNHCP) (2022). CAR (Chimeric Antigen Receptor) T-Cell Therapy: A Resource for Healthcare Professionals.

[ref-368138] European Blood and Marrow Transplantation Society (2023). Introducing the Immune Effector Cells Forum for Nurses.

[ref-368132] Advanced Therapy Treatment Centres (ATTC) (2020). Resources.

[ref-368135] Chen K., Wang S., Qi D., Ma P., Fang Y., Jiang N., Wu E., Li N. (2022). Clinical Investigations of CAR-T Cell Therapy for Solid Tumors. Frontiers in immunology.

[ref-368137] Dulan S. O., Viers K. L., Wagner J. R., Clark M. C., Chang B., Gorospe G. L., Londrc A., Brown A. S., Rosenthal J., Smith E. P., Forman S. J., Snyder D. S., Budde L. E. (2020). Developing and Monitoring a Standard-of-Care Chimeric Antigen Receptor (CAR) T Cell Clinical Quality and Regulatory Program. Biology of blood and marrow transplantation: journal of the American Society for Blood and Marrow Transplantation.

